# Uterine Epithelioid Trophoblastic Tumor in a 44-Year-Old Woman: A Diagnostic Dilemma

**DOI:** 10.3390/clinpract11030078

**Published:** 2021-09-13

**Authors:** Maria-Gabriela Aniţei, Diana-Elena Lazăr, Raluca Alina Pleşca, Cristina Terinte, Iulian Radu, Viorel Scripcariu

**Affiliations:** 1 1-st Surgical Oncology Unit, Regional Institute of Oncology, General Henri Mathias Berthelot 2-4, 700483 Iasi, Romania; raduiuli@gmail.com (R.I.); vscripcariu@gmail.com (S.V.); 2 Grigore T. Popa, Surgical Department, University of Medicine and Pharmacy, 700115 Iasi, Romania; 3 Oncology Department, Regional Institute of Oncology, 700115 Iasi, Romania; lazardianaelena@yahoo.com; 4 Radiology Department, Regional Institute of Oncology, 700115 Iasi, Romania; plesca_ralu@yahoo.com; 5 Pathology Department, Regional Institute of Oncology, 700115 Iasi, Romania; cterinte@gmail.com

**Keywords:** epithelioid trophoblastic tumor, pleomorphic leiomyosarcoma, vaginal bleeding

## Abstract

Background: Epithelioid trophoblastic tumor (ETT) is a rare and newly defined disease, which most commonly occurs in women of reproductive age and can be a sequela of any gestational event. ETT can be present in both intrauterine and extrauterine sites. Case report: A woman of reproductive age, without specific comorbidities and with a single pregnancy and natural childbirth eight years ago, was diagnosed initially with poorly differentiated pleomorphic leiomyosarcoma on the hemostatic uterine curettage. Conclusion: Our case highlights that ETT presents a diagnostic challenge due to its rarity and histologic resemblance to other pathologies. Misdiagnosis delays effective treatment and affects survival. To date, only 8 cases of ETT of the uterus without previous gestational event and normal human chorionic gonadotropin (β-HCG) levels in a 60-year literature survey have been reported.

## 1. Introduction

The ETT is a very rare form of gestational trophoblastic neoplasms (GTNs) that grows slowly, produces low levels of beta-subunit of human chorionic gonadotropin (β-hCG), presents late-onset metastatic capability and is resistant to different chemotherapy regimens [1]. In clinical practice, due to its rarity and large spectrum of clinical presentation and limited knowledge, ETT often goes misdiagnosed and, subsequently, mismanaged. An ETT may occasionally be misdiagnosed as a choriocarcinoma, placental site trophoblastic tumor, squamous cell carcinoma or other tumor before and after hysterectomy because microscopic ETT examinations share similarities with other tumors [2]. The patients generally present with vaginal bleeding, abdominal mass or amenorrhea. According to the medical literature, most ETTs have occurred in patients of reproductive age and were usually associated with a previous gestational event [1]. The most commonly, tumors develop from the uterine cervix or lower uterine segment. At present, no optimal treatment has been identified, an ETT does not appear to be as chemo sensitive as other GTNs. Although the prognosis is bad, early detection, diagnosis and treatment are important factors for patient prognosis.

## 2. Case Report

A 44-year-old woman (gravida 1, para 1, live 1), without specific comorbidities, was referred to the Emergency Department at a primary care facility for sudden onset vaginal bleeding, associated with pain of moderate intensity in the lower part of the abdomen. Her last menstrual period occurred two weeks before her presentation, and previous menstrual cycles were regular. She did not smoke or drink alcohol. The patient was otherwise fit and well, with no other significant medical history. She had no significant family history and she is married, with a 12-year-old daughter. The delivery was vaginal, uncomplicated, term pregnancy, without any known difficulties. Also, the patient denied intermenstrual bleeding, post-coital bleeding and recent changes in menstrual cycle.

The general condition of the patient appeared to be good. Bimanual examination revealed an anteverted uterus increased in size, firm consistency, painless. There were no masses palpated in the vaginal canal or adnexa. Abdominal-pelvic ultrasound showed an endometrial thickness of 8 mm and multiple hypoechoic intramural masses of different sizes. As the cause of bleeding could not be determined from any coagulation disorder or a scar site, a diagnostic and hemostatic uterine curettage was performed and histology showed poorly differentiated pleomorphic leiomyosarcoma.

In April 2020, the patient was submitted to our unit for treatment. After discussion at the oncology multidisciplinary team meeting (MTD), a second pathological opinion was requested.

**Preoperative investigations and surgical treatment**

Microscopic re-evaluation of slides in the biopsy curettage product was completed with the immunohistochemical examination that established the diagnosis of ETT.

**Figure 1:**
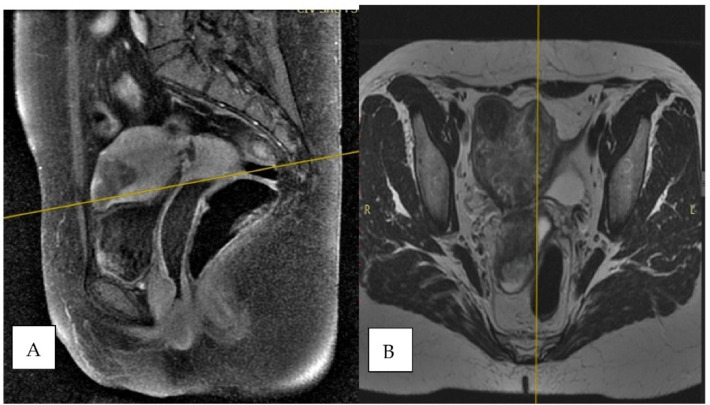
Radiological images of abdominal MRI scan with gadolinium (**A**) sagittal: perpendicular al coronal (FSE T1-FS), (**B**) axial oblique T2-weighted sequence showing large uterus with multiple heterogeneous masses centered within the uterine fundus.

Pelvic magnetic resonance imaging (MRI) with gadolinium showed the presence of a large uterus measuring 51 (longitudinal) × 79 (max anteroposterior) × 91 mm (transverse), with inhomogeneous structure due to a fundic tumor mass measuring 23 × 32 mm with moderate hypo signal T1, with 2/3 myometrial invasion. The right ovary had multiple peripheral cysts measuring 17 × 12 mm (Figure 1).

For the systemic evaluation of the patient, a thoraco-abdominal CT-scan was performed which showed the absence of lesions suspected of secondary determinations.

The images and tumor results had been reviewed at the oncology MTD, and the recommendation was surgical treatment. 

Laboratory tests showed mild neutrophilic leukocytosis (leukocytes—10,100 mm^3^, neutrophils—8160 mm^3^) and normal β-HCG (<2 ng/mL), α-fetoprotein (AFP) and CA125 levels (18.4 UI/mL).

**Figure 2:**
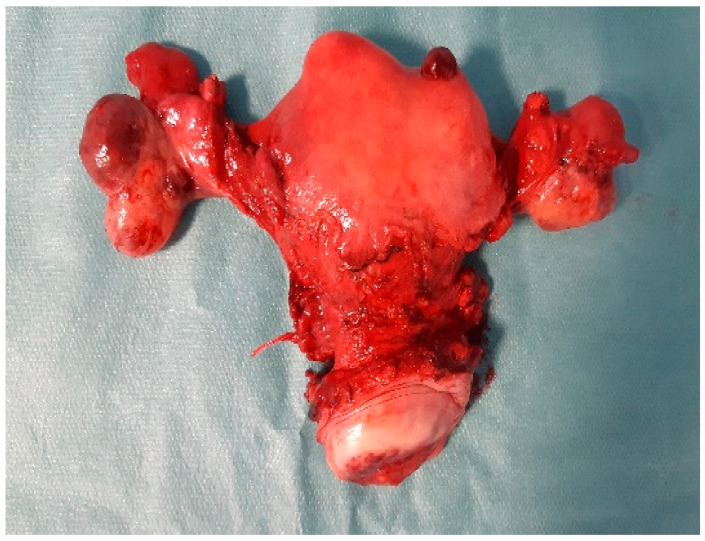
Operative image: Macroscopic examination of the uterine tumors with extension on the left horn.

After preoperative preparation was done, radical hysterectomy with bilateral salpingo-oophorectomy and bilateral pelvic lymphadenectomy (level I and II) was performed (Figure 2). The procedure was uncomplicated. The patient has had a good recovery after surgery and she was discharged on day 5 postoperatively.

**Anatomopathological evaluation of the surgical specimen**

The **macroscopic** evaluation showed the uterus with irregular contour. The uterine cavity is 4 cm long. At the level of the uterine fundus, with extension to the left horn, there were noted a tumor mass of 3.7/2.5/2 cm, well delimited, pearly white, fasciculate appearance and friable consistency. The tumor protrudes in the uterine serosa and infiltrates over 50% of the thickness of the myometrium. In the right hemibody of the uterus, intramural, there were found multiple nodular tumors, the largest being 2.3 cm in diameter, well defined, pearly white, fasciculate appearance and elastic consistency. The right ovary measured 4.2 cm × 5.1 cm × 1.7 cm with unilocular sero-cytrin ovarian cyst measuring 2.2 cm. The left ovary measured 2.9 cm × 2.2 cm × 0.8 cm with two unilocular sero-cytrin ovarian cysts measuring 0.8 cm.

The **microscopic** evaluation of the surgical specimen showed a uterine nodular tumor with dimensions of 3.5 cm × 2.5 cm × 2 cm developed in the uterine fundus area with extension to the left horn, apparently well delimited both macroscopically and microscopically—*pushing type margin*.

**Figure 3:**
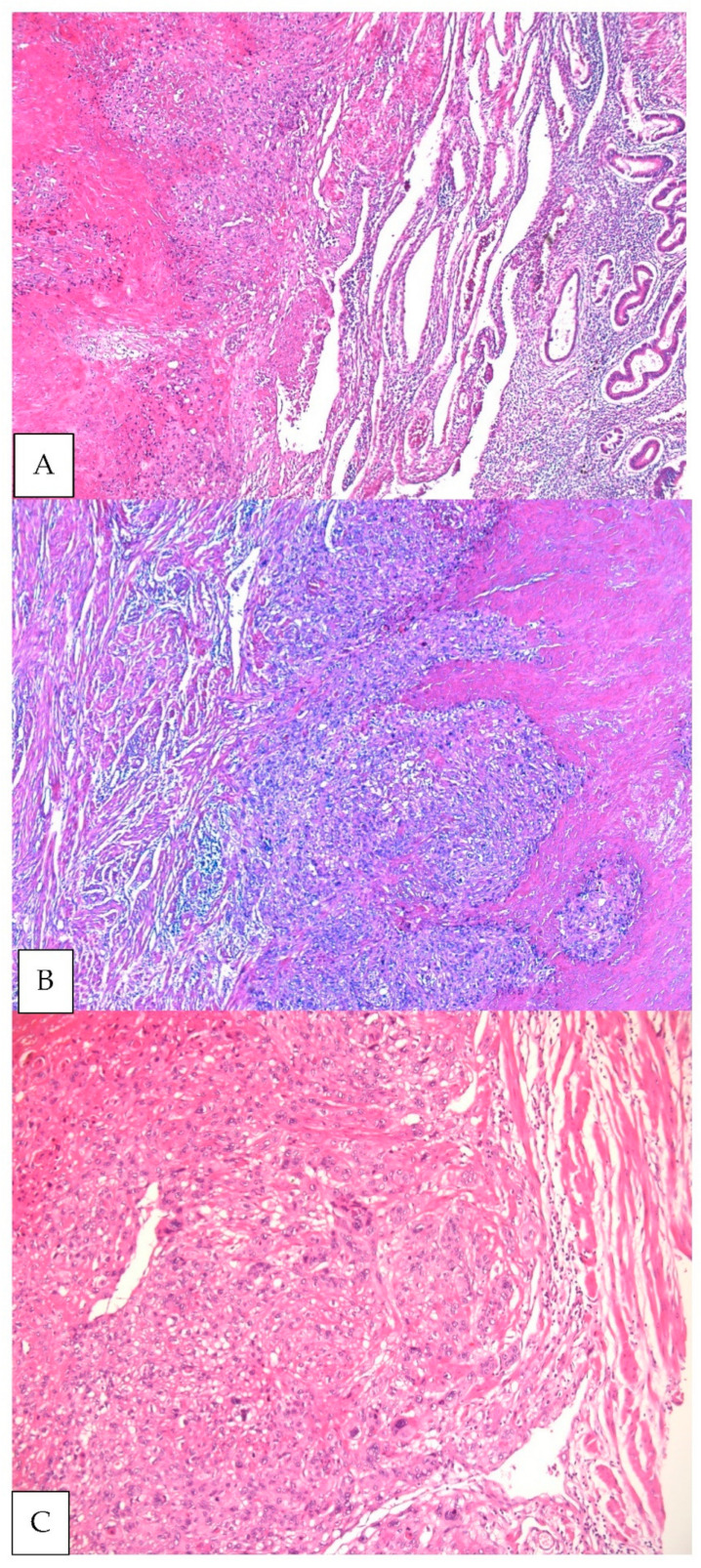
Histological analysis: hematoxylin-eosin stain; (**A**) tumor infiltration of the endometrial mucosa (magnification ×5); (**B**) pushing border and tumor necrosis (magnification ×5) (**C**) cellular atypia and tumor cells lining the blood vessel (magnification ×10).

Intermediate trophoblast tumor cells are arranged in a fibrous eosinophilic material with vesicular nuclei. Tumor cells surround and sometimes completely replace the vascular walls in some medium-sized vascular spaces. Numerous atypical mitoses are identified—19 mitoses/10/high power field. At the level of the uterine serosa, near the tumor formation, an area of its rupture is identified with the exteriorization on the serosa of a small island of tumor cells accompanied by necrosis. The specimen margins were, notably, free of tumor (Figure 3). All the pelvic lymph nodes are with a preserved structure, without tumor metastases.

**Figure 4:**
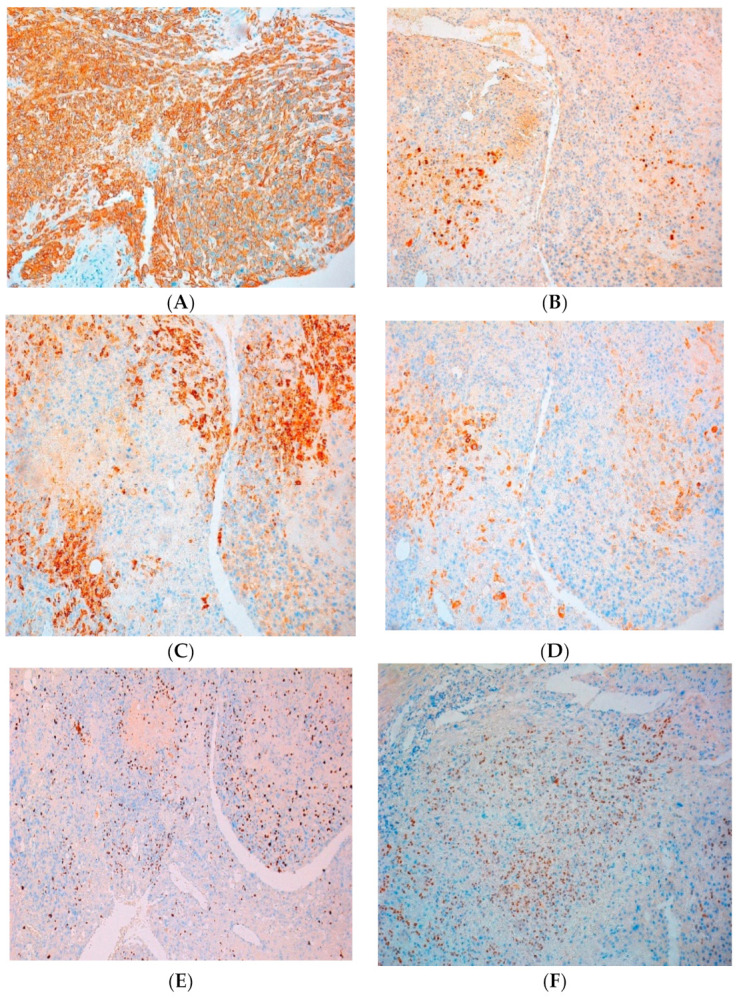
Immunohistochemistry assay: (**A**) CK-AE1/AE3 positive (×10) (**B**) focal positive cycline D1 (×10) (**C**) EMA positive (×10) (**D**) focal positive inhibin alpha (×10) (**E**) The Ki-67 index revealed 25% on proliferation activity (×5). (**F**) P 63-weakly positive, zonal extended in tumor cells (×10).

**Immunohistochemistry** staining of the tumor cells revealed SMA was negative in tumor cells, with positive internal control, desmin was negative in tumor cells with positive internal control, CKiT negative in tumor cells and positive in mast cells, CD 10 intense and diffuse positive in tumor cells and p63 weakly positive, zonal extended in tumor cells (Figure 4). The histology diagnostic was an epithelioid trophoblastic tumor of the uterus, pT1N0M0.

The FIGO prognostic score is 8, with high risk (age = 1, historical-term pregnancy = 2, interval of the previous pregnancy = 4, bHCG level = 0, tumor size = 1, no metastases = 0).

Sixteen months postoperatively, the patient remains disease free according to the paraclinical investigations. She is being monitored monthly with serum β-HCG.

## 3. Discussion

Shih and Kurman first described this pathology in 1998 and named it ETT, distinct from placental site trophoblastic tumor (PSTT), which is believed to arise exclusively from chorionic-type intermediate trophoblast [2]. The World Health Organization classified ETTs as a form of GTN in 2003 [3]. ETT is far less common than PSTT.

The incidence of ETT after a term pregnancy is 1:150,000 pregnancies, with evidence of ethnic variation, representing 1–2% of GTN cases [4,5].

In most of the patients, as in our patient, ETT usually occurs in women of reproductive age, after a full-term delivery. Rarely, it can occur in postmenopausal women. The interval between the ETT and the antecedent pregnancy ranges from several months to many years. Like PSTTs, the antecedent pregnancy like in our case is more often female. The gestational history of our case was represented by full-term delivery. Serum β-HCG levels were almost always raised at the time of diagnosis, Ref. [2] which is not consistent with our case.

To date, only 8 cases of ETT of the uterus with normal serum β-hCG in a 61-year literature survey (PubMed 1961–2021) have been reported [6,7,8,9,10,11,12,13] (Table 1). Our case is the 9th case in literature, to the best of our knowledge, to present with normal β-hCG levels. 

On macroscopic examination, ETT almost always presents itself as a discrete solitary nodule of up to 5 cm, with well-circumscribed border, with solid, tan to brown surface, often with areas of hemorrhage and necrosis located in the fundus (as in our patient), lower uterine segment, endocervix or lung. Rare cases have included pulmonary [14] and abdominal wall ETTs [15] without an apparent uterine lesion. From the published material, the survival rate is nearly 100% for non-metastatic cases confined to the uterus, but decreases to 50–60% in patients with metastasis [16].

The correct diagnosis can only be made following histological examination and confirmed by immunohistochemistry. The most important and difficult part is to distinguish ETTs from PSTT—which may be challenging due to non-specific clinical and biological features, which frequently suggest an ectopic pregnancy. Useful clinical features in the distinguishing diagnoses PSTT and ETT include: vaginal bleeding in ETTs in comparison with abortion and amenorrhea in cases of PSTT.

The primary diagnosis of our patient was poorly differentiated pleomorphic leiomyosarcoma. In our case, we took the sequential steps in differentiating diagnoses as presented previously. Central histology review by pathologists with GTN’s expertise is crucial to minimize misdiagnosis, because it may be difficult to differentiate PSTT and ETT from each other, and other types of GTN’s.

Microscopically, the tissue from PSTTs have a more infiltrative myoinvasive pattern, an increased sheet-like growth (versus nests and cords), larger cells with more nuclear pleomorphism and multinucleation, prominent and distinctive vascular invasion, strong diffuse staining for hPL and Mel-CAM (CD146), but negative staining for p63 and p40. Extensive or ‘geographic’ necrosis is often present in ETT. Moreover, PSTT is p63 and p40 negative. In consideration to PSTT, indicated above, ETT has shown staining of PD-L1. These results indicate that PD-L1 positive ETTs may gain a benefit from immune checkpoint inhibitor therapy [17,18].

Immunohistochemical analysis for our case is summarized in Table 2, with a comparison between the most important immunohistochemistry panel of PSTT and ETT, according to WHO Classification of Tumours [19].

However, reviewing the available literature, a few risk factors generally appear to be associated with favorable or unfavorable outcomes. Time from antecedent pregnancy > 4 years, a high mitotic rate > 6/10 HPF, atypia, vascular invasion, myometrial invasion beyond the inner one-third, stage III or IV, diffuse uterine multifocal disease have been associated with worse outcomes for women with ETT [20,21]. The FIGO anatomical stage is a significant prognostic factor for ETT [22].

Overall, this case is a stage I disease (p T1N0) with some good prognostic factors, but at least two poor prognosis factors (19 mitoses/10/HPF, antecedent pregnancy > 4 years), which make the prognosis not completely clear (prognostic score = 8). Due to this features, surgery is the most suitable treatment, usually total hysterectomy with or without bilateral salpingo-oophorectomy [23].

A series of recent studies [24,25,26] have shown that patients with disease limited to the uterus may be cured by primary hysterectomy without adjuvant chemotherapy. Combined surgery plus chemotherapy are recommended for patients with higher β-hCG levels and metastatic disease [26].

The present case underlines the difficulties experienced in diagnosing and treating ETT and highlights the importance of reporting any new cases to add to the understanding of this unusual disease and to achieve a more stable approach for managing these patients.

## 4. Conclusions

The epithelioid trophoblastic tumors are rare and aggressive malignant uterus tumors, with particular diagnostic challenges, especially when they occur at a long distance from the pregnancy. The immunohistochemical examination of the uterine curettage product may establish the preoperative diagnosis of ETT. It is essential that the clinician be familiar with the lesion, and be anticipative to the possible presence of this disease in order to make early diagnosis.

The authors’ opinion is that ETT would be best treated in a tertiary center by an experienced multidisciplinary team.

## Figures and Tables

**Table 1:** Clinical data and management for cases in the literature with ETT and normal β-hCG levels.

Case No.	Publication	Age	Recent Gestation History	Interval to Antecedent Pregnancy	Maximum Serum β-hCG	Symptom	Management
1	Vencken et al. [6]	43	Abortion	13 years	<2	Amenorrhoea	surgery
2	Chen et al. [7]	42	Term	1 year	1 week after surgery	Vaginal Bleeding	surgery
3	Kim et al. [8]	35	Term	-	N	Abdominal pain	surgery
4	Scott et al. [9]	44	Term	4 years	N	Abdominal pain	surgery
5	Yang et al. [10]	39	Term	9 years	N	Abdominal mass	surgery and adjuvant chemotherapy
6	Almarzooqi et al. [11]	47	–	–	N	Vaginal Bleeding	surgery
7	Stănculescu et al. [12]	35	Abortion	10 years	N	Vaginal Bleeding	surgery
8	McGregor et al. [13]	68	Term	30 years	N	Vaginal Bleeding	surgery

**Table 2:** Summary of immunohistochemical stains ETT versus PSTT with results for the case [19].

Immunohistochemical Stain	PSTT	ETT	Results for Our Case
CD10	positive	positive	intense and diffuse positive in tumour cells
p63	negative	positive	positive, zonal extended in tumour cells
CKAE1/AE3	positive	positive	diffusely positive in tumour cells
EMA	positive	positive	positive in focus in tumour cells
Ki67	5–10%	>10%	positive in 50% of tumor cells
Cyclin D1	negative	positive	positive in focus in tumor cells
Inhibin alpha	negative	positive	focal positive in tumor cells

CKAE1/AE3 = cytokeratin AE1/AE3; EMA = epithelial membrane antigen.

## Data Availability

Data are available on request due to restrictions.
